# Anthocyanin-Functionalized Hydrophobic Cellulose Composite Films as Sensitive Colorimetric Indicators to Monitor Beef Freshness

**DOI:** 10.3390/foods14223944

**Published:** 2025-11-18

**Authors:** Xuemei Cai, Changqiu Li, Yujie Mo, Mingfeng Qiao, Jun Xiang, Shuang Wang, Meifeng Li

**Affiliations:** 1 Cuisine Science Key Laboratory of Sichuan Province, Sichuan Tourism University, Chengdu 610100, China; cxm121517@163.com (X.C.); lcq66988@163.com (C.L.); 18481233522@163.com (Y.M.); mfqiao@163.com (M.Q.); 2 College of Biomass Science and Engineering, Sichuan University, Chengdu 610065, China; 85401296@163.com; 3 State Key Laboratory of New Textile Materials and Advanced Processing, Wuhan Textile University, Wuhan 430200, China; limeifeng2707@163.com

**Keywords:** nanocellulose composite film, hydrophobic, anthocyanin, visual indicators, antibacterial, biodegradable

## Abstract

In recent years, emerging biodegradable food-packaging materials based on nanocellulose with excellent comprehensive performance have become a promising, environmentally friendly raw material, which can be used as an ideal substrate for degradable composite films. However, the poor water resistance and flexibility of nanocellulose composite films limit their practical applications. Herein, nanocellulose films composed of chitin nanocrystals (ChNCs) and anthocyanin (BA) were utilized to obtain water-resistant, antibacterial, and flexible nanocellulose composite films. Owing to the nonpolar aromatic rings in the structure of BA, the water contact angle (WCAs) test indicated that the introduction of BA effectively improved the hydrophobicity of the nanocellulose composite film (WCA, up to 95°), and nanocellulose composite films possessed enhanced antibacterial/antioxidant properties (DPPH scavenging rate, 79.15%; 66.62% for *E. coli* and 78.07% for *S. aureus*). Importantly, the introduction of BA effectively enables the film to indicate the freshness of a nanocellulose food wrap, considering its pH/NH_3_-responsive properties. In addition, the weight loss rate of CCBA-9 film reached 90.21% after 13 days, demonstrating good biodegradability. Therefore, by innovatively introducing the synergistic system of BA and ChNCs, the material breaks through the technical bottleneck of traditional polymer packaging materials and provides a disruptive solution for the development of the next generation of environmentally friendly smart food packaging materials.

## 1. Introduction

The widespread use of contemporary petroleum-based food-packaging films has led to severe environmental pollution due to their persistent non-biodegradability. In this regard, renewable biomass has garnered substantial research interest due to its intrinsic biocompatibility, biodegradability, and cost efficiency [1]. Cellulose nanofibers (CNFs), recognized as the most abundant natural polymer on Earth, exhibit exceptional mechanical robustness, tunable surface chemistry, and biocompatibility [2,3,4]. These characteristics make CNF a cornerstone material for next-generation sustainable packaging systems for degradable composite films. However, the poor mechanical properties of nanocellulose composite films limit their practical applications.

At present, the development of food preservation based on nanocellulose composite films has made great progress [5,6,7]. Several strategies have been developed to improve the mechanical performance of nanocellulose food wraps, including plasticization [8], flexible polymers [9], cross-linking toughening [10,11,12], and multiscale microstructure [13]. Among them, nanocomposite enhancement is generally regarded as one of the most effective ways to improve the mechanical performance of nanocellulose composite films. Chitin nanocrystals (ChNCs), the main structural polysaccharide extracted from arthropods, are the second most abundant natural polymer on earth and possess properties, such as biodegradability, nontoxicity, and antimicrobial properties [14,15]. Nevertheless, the method of blending inorganic nanomaterials can significantly improve high tensile strength and Young’s modulus of the nanocellulose composite films, but the rigidity of inorganic nanomaterials causes drawbacks, such as interface incompatibility, which leads to decreased mechanical properties, fragile continuous stress, and shortened service life. Therefore, to meet the requirements of the mechanical properties and interface compatibility of the nanocellulose composite films, it is vital to improve the interfacial stability of the nanocellulose composite-based plastic wrap materials while ensuring their high mechanical strengths.

Inspired by plant polyphenols, anthocyanins are a kind of plant secondary metabolites with natural safety, chromogenic, antibacterial/antioxidant, and biological regulation functions, whose chemical structure diversity and pH response characteristics make them valuable for important applications in food-packaging fields [16,17,18]. The addition of anthocyanins not only endows the film with excellent antioxidant and antibacterial properties but also forms interaction forces with most biomacromolecular polymers and enhances other physical and chemical properties of the active film. More importantly, the unique characteristic of chromogenic endows the nanocellulose composite film with the ability to perform colorimetric determination, which could be used for food preservation. In this way, it is necessary to make full use of the multiple interface interactions between anthocyanins and nanocelluloses to prepare with interface stability, food preservation, and antibacterial/antioxidant properties [19].

In this work, ChNCs and anthocyanins are introduced into nanocellulose networks as raw materials successively to construct solid and antibacterial/antioxidant nanocellulose composite films with chromogenic properties. Afterwards, the mechanical properties, water resistance, and oxygen resistance of nanocellulose composite films were studied. The effects of different BA contents on the antibacterial/antioxidant properties of the nanocellulose composite films were analyzed. Different ChNCs/BA-based composite films were subjected to food preservation experiments. The freshness of the food was evaluated through various analytical methods, including visual observation, weight loss rate testing, hardness testing, soluble solids testing, and ascorbic acid testing, leading to a value assessment of the preservation effect of the composite film. This provides a biodegradable food preservation active packaging film that can replace traditional non-degradable plastics, reducing long-term pollution to the environment.

## 2. Materials and Methods

### 2.1. Materials

TEMPO-oxidized cellulose nanofibers (CNF, 1.0 wt%) were purchased from Shanghai Scienck Co., Ltd. (Shanghai, China). Chitin nanocrystals (ChNCs, 0.6 wt%) were purchased from Beijing Beifang Tianchen Technology Co., Ltd. (Beijing, China). Anthocyanin (BA) was obtained from Sigma-Aldrich Chemical Industry Co., Ltd. (Shanghai, China). In addition, deionized (DI) water was house-made. All ingredients were used without further purification.

### 2.2. Preparation of ChNCs-Incorporated Nanocellulose Composite Films (Labeled as CNF-ChNCs Composite Films)

CNF was stirred for 30 min and dissolved in deionized water to obtain a uniform CNF solution. After that, ChNCs were put into the above CNF solution to obtain a CNF/ChNCs mixture according to a CNF/ChNCs ratio of 1:1 (*w*/*w*). The total dry solid content for each film was fixed at 0.2 g. Eventually, the nanocellulose composite films were fabricated by casting the mixture on a 10 × 10 cm PTFE mold at ambient temperature for 7 days and then dried in a vacuum oven at 60 °C for 24 h before characterization. Moreover, the synthesis method of nanocellulose films without ChNCs (labeled as CNF films) is the same as CNF-ChNCs composite films.

### 2.3. Preparation of BA/ChNCs-Incorporated Nanocellulose Composite Films (Labeled as CCBA-x Composite Films)

**Figure 1:**
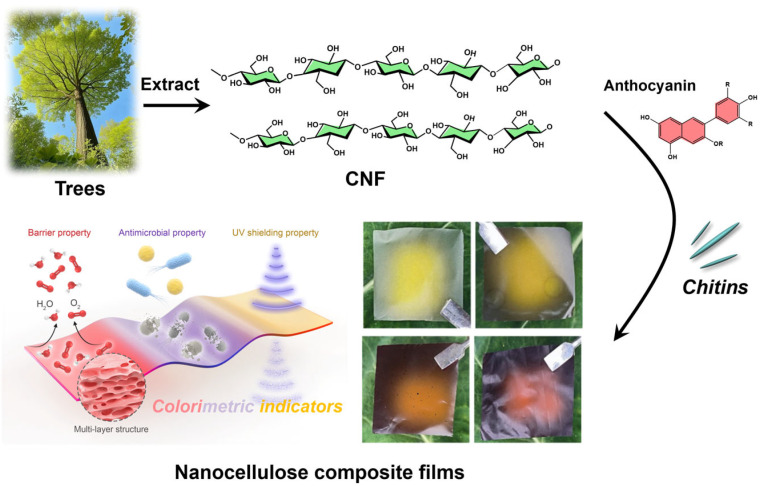
Schematic illustration of the preparation of nanocellulose composite films.

To prepare the blueberry anthocyanin (BA)-incorporated composite films (designated as CCBA-x), different loadings of BA (1.0, 3.0, 6.0, and 9.0 wt%) were introduced into the CNF/ChNCs mixture. The mixtures were magnetically stirred for 30 min to achieve homogeneity and then cast onto PTFE molds. The cast films were first dried at ambient temperature for 7 days, followed by further drying in a vacuum oven at 60 °C for 24 h. The detailed compositions and codes for all film samples are summarized in Table 1, and a schematic illustration of the film preparation process and its application is provided in Figure 1.

### 2.4. Characterization

#### 2.4.1. Structural Characterization

Ultraviolet–visible spectroscopy (UV-vis) was investigated, ranging from 800 to 200 nm, using an Analytic-Jena Specord S600 (Analytik Jena AG, Jena, Germany) instrument. Scanning electron microscopy (SEM) employed an Apreo S HiVoc scanning electron microscope (Thermo Fisher Scientific, Waltham, MA, USA) to analyze the cross-section morphologies of composite films. In addition, the samples were electrically conductive by sputtering with gold.

#### 2.4.2. Transparency and Stability Assay

The optical characteristics of the films were evaluated through visual observation and spectrophotometric measurements. For visual assessment, films were placed over a yellow floral background to demonstrate their transparency. UV-vis transmission spectra (200–800 nm) were recorded using a spectrophotometer (Shimadzu, Japan), with film specimens of 8 × 30 mm strips [20]. Film transparency was quantified based on the absorbance value at 600 nm. The opacity values were calculated as follows [21]:

$$\mathrm{Opacity}=(-\log_{10}T_{600})/d$$

where T_600_ is the transmittance at 600 nm, and d is the film thickness (mm), which was measured using a thickness gauge (GOTECH, Guangzhou, China).

To assess storage stability, film samples with a duplicate size of 2 cm × 2 cm were stored under ambient conditions (25 °C) for 21 days. The CIE lightness (L*), redness (a*), and yellowness (b*) components were monitored weekly using a portable colorimeter (NR10QC, 3nh, Shenzhen, China). The total color difference (*ΔE*) was calculated as follows:

$$\Delta E=\sqrt{{\left(L^{*}-L_{0}\right)}^{2}+{\left(a^{*}-a_{0}\right)}^{2}+{\left(b^{*}-b_{0}\right)}^{2}}$$

where $L_{0}$, $a_{0}$, and $b_{0}$ are the color parameters of the standard white plate, and L*, a*, and b* are the color parameters of the films.

#### 2.4.3. Water Contact Angle (WCA) Test

The water contact angles of composite films were determined using an optical contact angle goniometer (T200-Auto3 Plus, Biolin, Sweden). Each film was tested three times for accuracy.

#### 2.4.4. Moisture Content (MC), Degree of Swelling (DS), and Hygroscopicity (HC)

Determination of MC, DS, and HS was adapted from Zhang et al. [20]. Films were cut into 2 cm × 2 cm squares (m_1_), oven-dried to constant weight (m_2_), then immersed in 20 mL deionized water for 9 min. Photographs were taken at 3, 6, and 9 min. Surface moisture was removed with filter paper before weighing (m_3_). Separately, oven-dried films were conditioned at 95% relative humidity for 24 h and weighed (m_4_). MC, DS, and HC were calculated using the equations below:

$$\mathrm{MC}(\%)=(m_{1}-m_{2})/m_{1}\times 100\%$$

$$\mathrm{DS}(\%)=(m_{3}-m_{2})/m_{2}\times 100\%$$

$$\mathrm{HC}(\%)=(m_{4}-m_{2})/m_{2}\times 100\%$$

#### 2.4.5. Water Vapor Permeability (WVP) and Oxygen Permeability (OP)

The WVP and OP of the films were determined using a gravimetric method based on previous descriptions [22], with modifications for permeability calculation. Briefly, 20 mL vials with a neck diameter of 1 cm were prepared (exposed area = 0.0000785 m^2^). For WVP measurement, vials were filled with 3.0 g of anhydrous calcium chloride. For OP measurement, vials were filled with 5.0 g of deoxidizer. The vial openings were covered with film specimens and securely sealed. All sealed vials were placed in an environmental chamber (GHP-9270N, Shanghai Yiheng Scientific Instrument Co., Ltd., Shanghai, China) maintained at 25 °C and 80% relative humidity for 24 h. The WVP and OP of films were calculated:

WVP=(Δm×d)/(A×t×ΔP)

OP=(Δm×d)/(A×t×ΔP)

where Δm is the mass change (g) of the cup, d is the film thickness (mm), t is the equilibration time (day), A is the area (m^2^) of the cup mouth, and ΔP is the water vapor pressure difference across the film, which is measured as 2.22 kPa under the test conditions.

#### 2.4.6. Sensitivity to Ammonia

The ammonia responses of the films were evaluated using the method described by [23]. Ammonia solutions with concentrations of 0.1, 2.5, 5.0, 10, and 20 *v*/*v* were prepared. In total, 10 mL of each solution was transferred into a 15 mL centrifuge tube, with the film placed over the tube’s opening and secured with a rubber band. The tubes were then placed in a fume hood, and images were taken every 5 min using a mobile phone until no further significant color change occurred.

#### 2.4.7. Sensitivity to pH Solutions

The pH sensitivity of the films was evaluated following the method described by [23,24], with minor modifications. Briefly, 20 µL of blueberry anthocyanin solution (25 mg/mL) was added to 10 mL of aqueous buffer solutions (pH 1–14, adjusted with 0.01 M NaOH or HCl) and incubated for 20 min. Subsequently, 2 mL of each pH solution was applied onto the surface of 1 × 1 cm film samples and incubated for 10 min at room temperature. The color changes were recorded by taking pictures with a mobile phone.

#### 2.4.8. Antioxidant Activity

The antioxidant activity of the films was evaluated according to the DPPH radical scavenging assay [25]. Film samples (5 or 10 mg) were immersed in 4 mL of freshly prepared DPPH solution and incubated in the dark. Absorbance at 517 nm was measured at 10 min intervals (0–70 min) to calculate the free radical scavenging rate.

#### 2.4.9. Antibacterial Activity

This method is based on the approach used by [22,26], with slight modifications. Film samples (10 mg) were transferred aseptically into 15 mL centrifuge tubes and mixed with 1 mL of *E. coli* and *S. aureus* suspensions (10^8^ CFU/mL) separately. After 3 h of incubation at 37 °C, 10 mL of sterile saline was added, and the mixture was vortexed for 1 min. Serial dilutions were performed, and 100 μL of each dilution was plated onto LB agar. After 24 h of incubation at 37 °C, bacterial colonies were counted and photographed, with the no-film group (CK) serving as the control. Three replicates were used for each film.

### 2.5. Degradation Test

Square specimens (3 cm × 3 cm) of CNF, CNF-ChNCs, and CCBA-9 films were embedded in 40 g of natural soil, with the moisture content maintained at 15 ± 1%. The specimens were incubated under ambient conditions in Chengdu, China (June; diurnal temperature range: 20–30 °C). The films were photographed and weighed every 1–2 days. Prior to each measurement, the soil covering the films was carefully removed. Afterward, the films were re-covered with soil, and one pump of water was sprayed onto the soil surface using a 500 mL watering can. Monitoring continued until complete structural disintegration of the films was observed. The extent of degradation was quantitatively assessed by the percentage of mass loss, calculated using the equation below:

$$\mathrm{Weight}\mathrm{Loss}\mathrm{Rate}(\%)=(m_{0}-m_{t})/m_{0}\times 100\%$$

where m_0_ is the initial mass of the film (g), and m_t_ is the mass of the film during the degradation process.

### 2.6. Application in Beef Preservation

Fresh beef (10.0 g) purchased from the local market was placed in an uncovered transparent plastic box and sealed with CNF, CNF-ChNCs, and CCBA-9 films, respectively. A food-grade polyethylene (PE) film was used as the control, while the unsealed group served as the blank control (CK). All samples were stored at 25 °C. Every 12 h, three samples from each group were taken to measure the total viable count (TVC), total volatile basic nitrogen (TVB-N), weight loss, pH, and color.

## 3. Result and Discussion

### 3.1. Overall Design of the System

The inherent hydrophilicity of cellulose significantly limits the performance of cellulose films [10,11,12]. Therefore, enhancing their hydrophobicity and mechanical properties to achieve mechanical reinforcement in cellulose composites is a core research objective in this field. To address this challenge, we leverage the nano-reinforcement effect of chitin nanocrystals and the phenolic hydroxyl bridging capability of plant-derived anthocyanins, as observed in Figure 1. This approach enables multiple hydrogen bonding and nano-reinforcement modifications, successfully constructing novel cellulose nanocomposite films. This design not only improves the water vapor/oxygen barrier properties and water resistance of the films but also imparts pH and ammonia responsiveness. Eventually, chitin nanocrystal/plant polyphenol-modified nanocellulose composite films could be used to prepare food-packaging films with enhanced hydrophobicity and color-indicating functionality.

### 3.2. Structure Characterization

**Figure 2:**
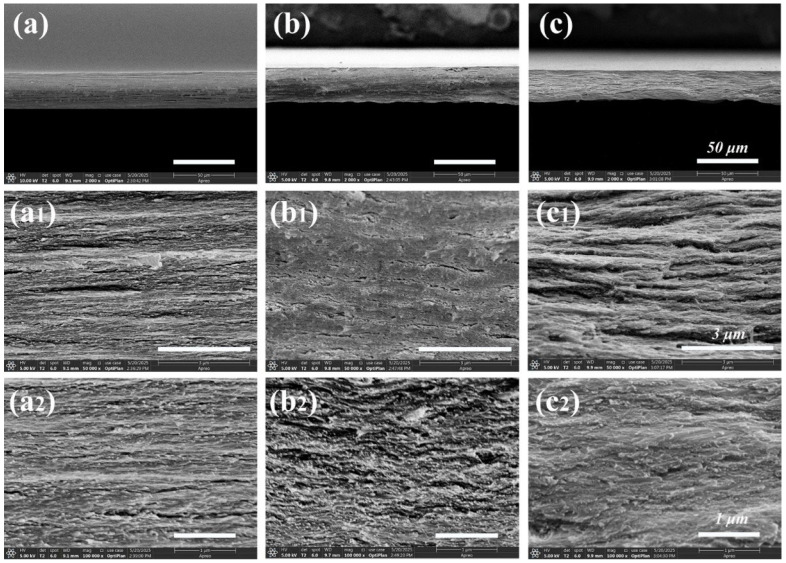
SEM images of CNF (**a**,**a1**,**a2**), CNF-ChNCs (**b**,**b1**,**b2**), and CCBA-9 (**c**,**c1**,**c2**) films at different magnifications (2000 times, 50,000 times, and 100,000 times).

The structural morphology of CNF, CNF-ChNCs, and CCBA-x films was examined using SEM, as observed in Figure 2. As seen in Figure 2a, the CNF film exhibited a more uniform layered structure. However, after the liquid nitrogen treatment, there were also many cracks in the sections of the CNF-ChNCs film, and the fibrous layers were twisted and brittle (Figure 2b), indicating that the interfacial stability of the nanocellulose films was destroyed by chitin nanocrystals. As shown in Figure 2c, the cross-section of the layered structure was covered by the fiber membranes to form a strong brick–concrete structure. These phenomena indicated that the addition of blueberry anthocyanins could effectively improve the interfacial compatibility between chitin nanocrystals and nanocellulose films.

**Figure 3:**
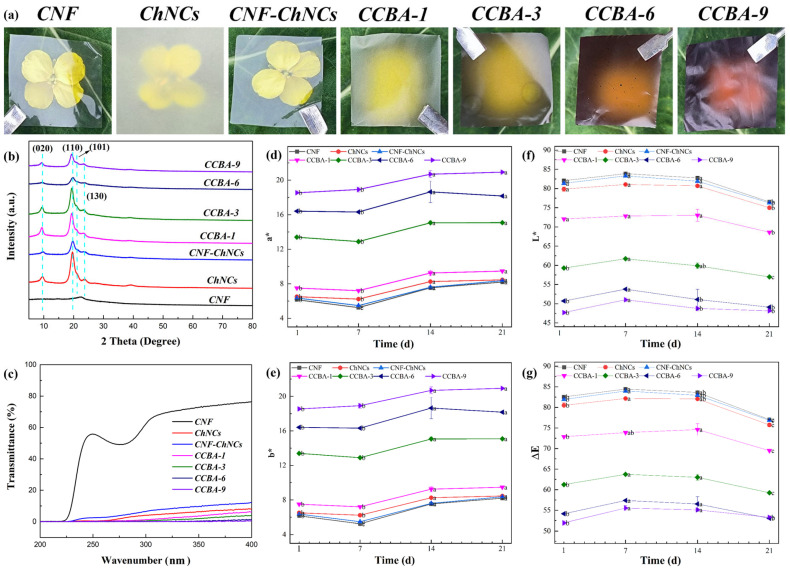
Structural characterization: (**a**) Digital photographs of CNF, ChNCs, CNF-ChNCs, and CCBA-x films; (**b**) XRD patterns; (**c**) UV-vis curves; (**d**–**g**) changes in a*, b*, L*, and *ΔE* values of the colorimetry of films at 25 °C.

XRD analysis was used to study the crystal structures of CNF, CNF-ChNCs, and CCBA-x films, as shown in Figure 3b. The representative intense diffraction peaks (2θ) of (020), (110), (101), and (130) for ChNCs appeared at 9.5°, 19.6°, 21.4°, and 23.6°. In addition, the characteristic peak of CCBA-x films shifted to a lower angle due to the incorporation of BA (at 2θ = 19.2°) [27]. Long-term exposure to ultraviolet (UV) light will accelerate the deterioration of packaged foods, so there is an urgent need to develop new degradable packaging materials with excellent barrier properties. As shown in Figure 3a, the CNF film exhibited high optical transparency. The UV resistance of CNF, CNF-ChNCs, and CCBA-x films was investigated using ultraviolet–visible spectroscopy, as shown in Figure 3c and Table S1. The UV absorbance of CNF-ChNCs increased from 23.9% to 91.8%, suggesting that chitin nanocrystals have a UV-blocking effect. The UV absorbance values of CCBA-x films were 93.9% (CCBA-1), 96.1% (CCBA-3), 98.8% (CCBA-6), and 99.4% (CCBA-9), indicative of superhigh ultraviolet absorption capacity of natural polyphenol BA. The incorporation of BA also influenced the films’ optical properties in the visible region. Specifically, the CCBA-9 film exhibited a very low transmittance (0.3%) at 600 nm, corresponding to a high opacity value of 109.87, which confirms that the film is highly effective at blocking visible light [21].

As anthocyanins increased, the color of the CCBA-x films changed from colorless to purple. Accompanied by significant increases in the color parameters a* (redness) and b* (yellowness), while L* (lightness) and *ΔE* (which refers to the testing unit for human eye perception of color difference in a uniform color sensation space) decreased synchronously (Figure 3d–g). During ambient storage (25 °C), the films displayed dynamic color behavior: a* and b* values decreased marginally by day 7, surged markedly at day 14 (*p* < 0.05), then declined progressively, while L* and *ΔE* exhibited transient increases at day 7 before continuous reduction. Notably, the *ΔE* values for CCBA-6 and CCBA-9 at day 21 showed no significant difference compared to their initial values (*p* > 0.05), whereas those of the other films decreased significantly (*p* < 0.05). This demonstrates that higher anthocyanin-loading enhances the color stability of the films, which may be attributed to the formation of an interlocked molecular structure at higher concentrations, improving environmental tolerance [28].

### 3.3. Water-Repellency Performance of CCBA-x Composite Films

**Figure 4:**
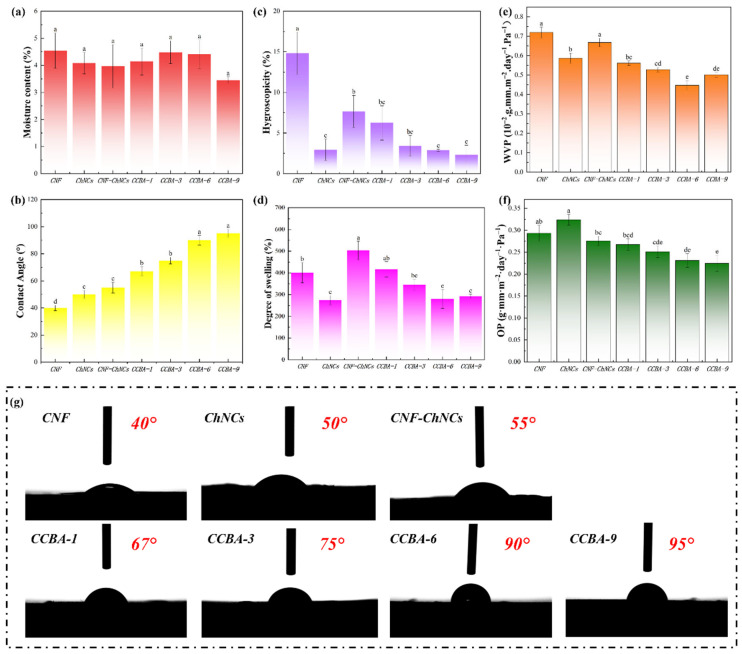
Water-repellency performance: (**a**) MC, (**c**) HC, (**d**) DS, (**e**) WVP, (**f**) OP, and (**b**,**g**) WCAs of CNF, ChNCs, CNF-ChNCs, and CCBA-x composite films, with lowercase letters indicating inter-sample variation, as determined by Tukey’s HSD test (*p* < 0.05).

The incorporation of ChNCs and BA exhibited a negligible impact on the equilibrium moisture content of the composite films (*p* > 0.05) (Figure 4a). This suggests that the bulk water absorption behavior was dominated by the hydrophilic CNF matrix, while the surface wettability could be independently modulated by the introduced components. Nevertheless, the hydrophilic nature of cellulose nanofibers undermined both the mechanical properties and structural integrity of cellulose films when exposed to water. To investigate the effect of ChNCs and BA on the hydrophobicity of the nanocellulose composite films, WCAs tests were used to measure the water resistance of CNF, CNF-ChNCs, and CCBA-x films, as shown in Figure 4b,g. Due to the large number of carboxyl groups, the WCA value of CNF films was 40°. With the addition of ChNCs, the WCA value of CNF-ChNCs films increased to 55°. Notably, the WCA value of CCBA-x films increased dramatically to 67° (CCBA-1), 75° (CCBA-3), 90° (CCBA-6), and 95° (CCBA-9), respectively. The ChNCs surface contains a large number of hydrophilic groups (hydroxyl and acetamide groups), resulting in a low WCA value. However, when ChNCs bind to anthocyanins, the hydrophobic benzene ring structure in the anthocyanin molecule could interact with the hydroxyl groups of ChNCs to form a hydrophobic surface, which reduces the direct contact area of ChNCs with water and significantly improves the hydrophobicity of the composite films.

This synergistic effect not only enhances hydrophobicity but also significantly improves bulk water-related properties. The hygroscopicity (HC) of CCBA-9 films was 2.31% at 95% relative humidity (RH), a decrease of 84.41% compared to the CNF film (Figure 4c). This decrease is attributed to the hydrophobic benzene rings of BA shielding the hydrophilic groups of ChNCs, effectively restricting water molecule access to the polar sites. Although CNF-ChNCs films exhibited a 25.54% higher degree of swelling (DS) than CNF due to interfacial incompatibility, CCBA-x films reduced DS via BA-mediated interfacial crosslinking, limiting polymer chain hydration; notably, CCBA-6 achieved a 30.27% lower DS than CNF (Figure 4d). Regarding the water vapor permeability (WVP), the addition of BA led to a nonlinear improvement. CCBA-9, due to its dense brick-mortar structure, shows a lower WVP value (0.50 × 10^−2^ g·mm·m^−2^·day^−1^·Pa^−1^) (Figure 4e). However, CCBA-6 exhibited the lowest WVP value (0.45 × 10^−2^ g·mm·m^−2^·day^−1^·Pa^−1^), which may be attributed to the high BA content causing nanopores that led to a slight rebound effect in the vapor channels. Regarding the oxygen permeability (OP), at 80% relative humidity, the OP of CCBA-9 gradually decreased to 0.22 g·mm·m^−2^·day^−1^·Pa^−1^ as the molecular barrier of BA increased (Figure 4f). Collectively, these enhancements extended food preservation efficacy by maintaining barrier integrity in humid environments.

### 3.4. Antibacterial/Antioxidant Properties of Nanocellulose Composite Films

**Figure 5:**
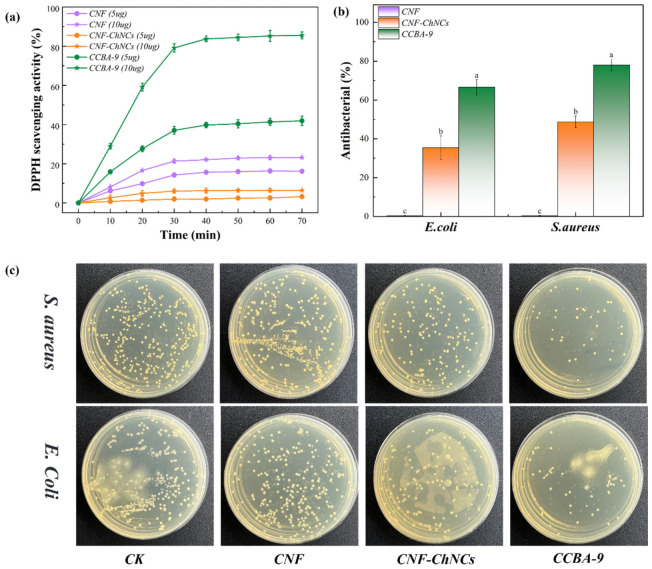
(**a**) DPPH-scavenging activity of CNF, CNF-ChNCs, and CCBA-9 films; (**b**) antibacterial rate, with lowercase letters indicating inter-sample variation, as determined by Tukey’s HSD test (*p* < 0.05); (**c**) antibacterial spectrum.

The principle of evaluating antioxidant capacity by the DPPH method is that DPPH can react with antioxidants. After the reaction, DPPH· loses this single electron and then undergoes a color change. Subsequently, the change in absorbance at a wavelength of 517 nm is measured to evaluate the antioxidant capacity of the antioxidants [29]. The scavenging effect of BA in CCBA-9 on DPPH is shown in Figure 5a. As seen in Figure 5a, the DPPH-scavenging rates of CNF (5 mg) and CNF (10 mg) films were 14.26% and 21.31%, respectively, after 30 min of reaction. However, the DPPH-scavenging rates of CNF-ChNCs (5 mg) and CNF-ChNCs (10 mg) films decreased to 1.97% and 6.01% due to a large number of carboxyl groups (-COOH) on the surface of TEMPO-oxidized cellulose, which acted as effective free radical scavengers (especially for free radicals such as DPPH and ABTS) to neutralize free radicals by providing hydrogen atoms or electrons. As observed, the DPPH-scavenging rate of CCBA-9 (5 mg) films increased to 37.03%, attributed to the introduction of natural antioxidants. Furthermore, the DPPH-scavenging rate of CCBA-9 (10 mg) films reached up to 79.15%, which was four times that of pure CNF films. These results demonstrated that the introduction of blueberry anthocyanins greatly improves the DPPH-scavenging activity of nanocellulose films.

The plate viable count method was used to evaluate the antibacterial properties of nanocellulose composite films. As observed in Figure 5b,c, the inoculation experiment on agar culture plates showed that the CNF films exhibited almost no antibacterial properties. However, the antibacterial rate of CNF-ChNC films was only 35.44% for *E. coli* and 48.75% for *S. aureus*. In contrast, the number of colonies on the CCBA-9 plate decreased significantly. In addition, the antibacterial rate of the CCBA-9 film was 66.62% for *E. coli* and 78.07% for *S. aureus*, indicative of the good antibacterial ability of blueberry anthocyanins. In this way, the above phenomena indicated that the nanocellulose composite films with the incorporation of plant polyphenol BA had excellent antibacterial/antioxidant properties, which could greatly prolong the storage life and enhance the stability of meats.

### 3.5. pH-Response and Ammonia-Sensitive Properties of CCBA-x Composite Films

**Figure 6:**
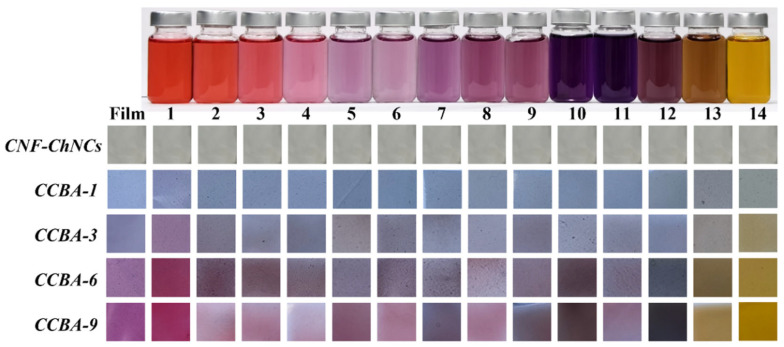
The pH-response performance of CNF-ChNCs and CCBA-x films in terms of color reaction to different pH solutions (pH range from 1 to 14).

**Figure 7:**
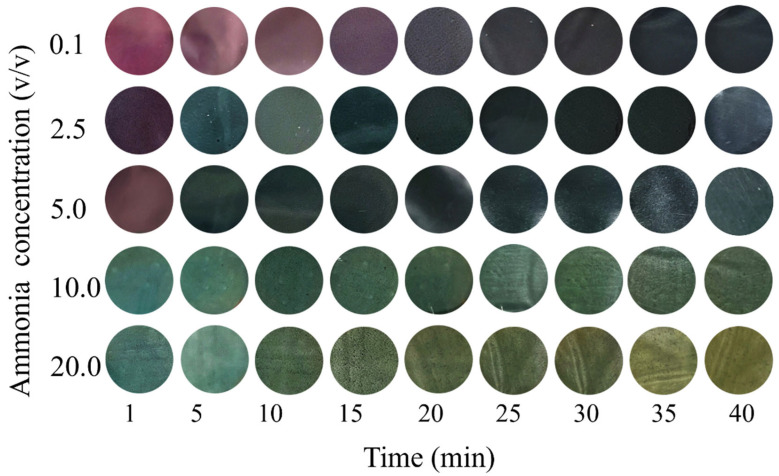
The ammonia-sensitive property performance of CCBA-9 films (exposure duration range from 1 to 40 min).

The behavior of CNF-ChNCs and CCBA-x films in terms of color reaction to different pH solutions was compared in Figure 6. CNF-ChNC films showed essentially no color reaction to different pH solutions. Notably, different CCBA-x films exposed to different pH solutions changed color. The higher the amount of anthocyanins, the more obvious the color change in the CCBA-x film after exposure to different pH solutions [30,31]. Concurrently, CCBA-x films exposed to different pH solutions changed their color from light blue to light gray (CCBA-1), from light purple to light yellow (CCBA-3), and from light violet to yellow (CCBA-6). Eventually, CCBA-9 films exposed to different pH solutions changed their color from violet to dark yellow, which is consistent with the response of anthocyanins to different pH solutions. Furthermore, the time-dependent color values for CCBA-x film toward ammonia are displayed in Figure 7. It is seen that the brightness of CCBA-x film exhibits a tendency to steadily fall with increases in the time of exposure to ammonia. Meanwhile, when the ammonia concentration is 0.1 *v*/*v*, the color of the film also changes significantly from purple to violet, indicating its apparent color variations that can be distinguished by human eyes [32]. These observations suggest that the CCBA-x film possesses a good color response toward ammonia and hence holds huge promise for use in smart food packaging.

### 3.6. Food Preservation

**Figure 8:**
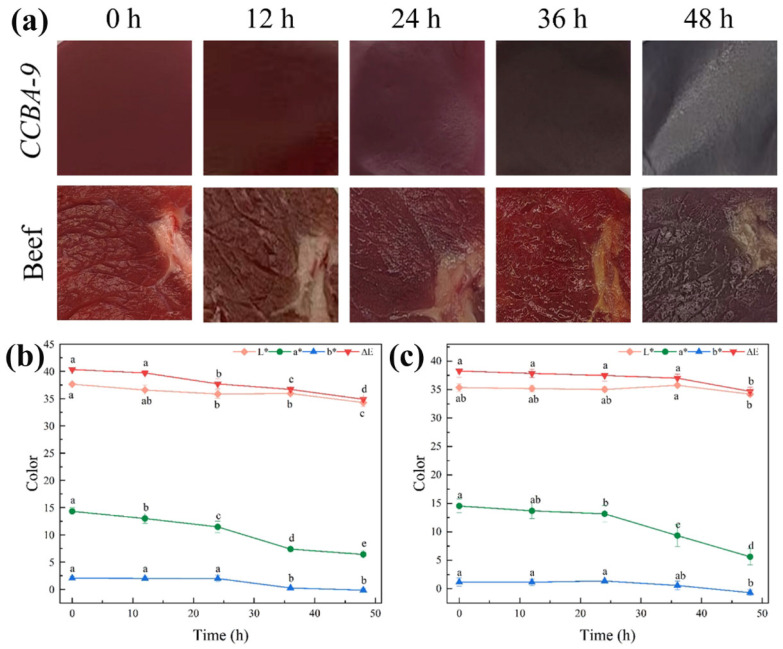
(**a**) Appearance of the film and beef after being stored in the CCBA-9 group for 48 h; (**b**) color change of beef; (**c**) color change of CCBA-9 film, with lowercase letters indicating variations at different time points, as determined by Tukey’s HSD test (*p* < 0.05).

This study evaluated the preservation and indication effects of CCBA-9 film for beef stored at 25 °C, using an accelerated shelf-life test to rapidly assess film functionality. PE film and unpackaged controls (CK) served as comparisons (Figure 8, Table 2). During storage, beef undergoes oxidation and microbial proliferation, leading to the formation of alkaline nitrogenous compounds (such as ammonia and amines) from protein degradation [33]. All samples initially exhibited a decrease in pH, followed by an increase, primarily due to lactic acid production from glycogen breakdown. The pH of the CK group remained lower than that of the packaged groups, as it experienced significant water loss, which concentrated acidic metabolites and promoted the growth of acid-producing bacteria. The PE group exhibited the lowest weight loss due to its superior physical barrier properties.

Regarding TVB-N and TVC, CCBA-9 demonstrated its preservation potential by maintaining the lowest values for both parameters, attributed to its antibacterial and antioxidant functions. Under acidic conditions, cationic anthocyanins disrupted the integrity of Gram-positive bacterial cell membranes, keeping the TVC at 4.36 lg CFU/g within 24 h, which was significantly lower than that of the CK group. Additionally, the antioxidant properties of anthocyanins delayed the accumulation of TVB-N, which was 8.51 mg/100 g at 24 h, 40% lower than that of the CK group. After 36 h, TVB-N and TVC values for all groups exceeded the spoilage threshold (TVB-N > 15 mg/100 g, TVC > 6 log CFU/g). At this stage, the pH of the CCBA-9 group increased to 6.47, leading to reduced anthocyanin activity, yet the film’s indicator function remained effective. Its color transitioned from purple–red at 24 h to dark black at 36 h (Figure 8a).

The a* value of CCBA-9 exhibited significant changes throughout the storage period and was highly correlated with key spoilage parameters, TVB-N and TVC (r = 0.988, *p* < 0.01; r = 0.976, *p* < 0.05) (Figure 8b). These results indicate that CCBA-9 successfully facilitated real-time visual monitoring of the spoilage process.

### 3.7. Whole Life Cycles of CCBA-x Composite Films

**Figure 9:**
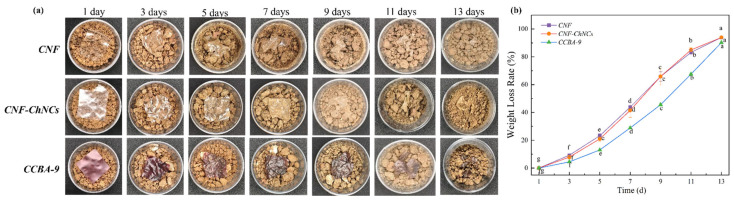
Appearance (**a**) and detailed data (**b**) of the CNF, CNF-ChNCs, and CCBA-9 films during whole life cycles, with lowercase letters indicating variations at different time points, as determined by Tukey’s HSD test (*p* < 0.05).

Considering the significant environmental harm caused by traditional non-degradable polymer films, the degradability is crucial for the environmental sustainability of food wraps [34,35]. To investigate the degradability of the composite films, CNF, CNF-ChNCs, and CCBA-9 films were buried in natural soil to observe and record the disintegration residuals of composite films, as shown in Figure 9. As observed in Figure 9a, all samples disintegrated gradually over time. After 13 days, no visible film fragments remained in the soil. As depicted in Figure 9b, the weight loss of CNF films reached 93.89%, demonstrating excellent biodegradability. At the same time, the weight loss of CNF-ChNCs films reached 93.88%. Moreover, weight loss rates of CCBA-9 films reached 90.21% at 13 days. These results prove that the nanocellulose composite film has excellent biodegradability and degrades in natural environments, making it highly suitable for large-scale applications in food packaging.

## 4. Conclusions

In this study, a multifunctional nanocellulose composite film was fabricated by introducing ChNCs and anthocyanins successively into the nanocellulose networks. These nanocellulose composite films exhibited good water resistance (WCA, up to 95°) and oxygen resistance. In addition, the introduction of BA could significantly enhance the antibacterial/antioxidant properties of the cellulose films (DPPH scavenging rate, 79.15%; 66.62% for *E. coli*, and 78.07% for *S. aureus*). Moreover, the freshness of the food was evaluated through multiple analytical methods, including visual observation, weight loss rate determination, hardness determination, soluble solids determination, and ascorbic acid determination. Importantly, the introduction of BA effectively enables the film to indicate the freshness of the nanocellulose food wrap, considering its pH/NH_3_-responsive properties. Due to the good biodegradability of various bio-based components in CCBA-x films, the weight loss rate of the CCBA-9 film reached 90.21% after 13 days, which is beneficial for environmental sustainability. Overall, this work presents a convenient and cost-effective solution for preparing biodegradable, antibacterial, and water-resistant cellulose composite films with continuous food freshness monitoring, making it ideal for long-term food preservation.

## Figures and Tables

**Table 1:** Composition and coding of the prepared composite films.

Sample Code	CNF/ChNCs Matrix (Dry Mass Ratio)	BA (wt%)
CNF	100/0	0
ChNCs	0/100	0
CNF-ChNCs	50/50	0
CCBA-1	50/50	1
CCBA-3	50/50	3
CCBA-6	50/50	6
CCBA-9	50/50	9

**Table 2:** Changes in various performances of beef in CK, PE, and CCBA-9 groups during storage.

	Time (h)	CK	PE	CCBA-9
pH	0	5.67 ± 0.02 bcA	5.67 ± 0.02 bcA	5.67 ± 0.02 cA
	12	5.48 ± 0.1 cA	5.53 ± 0.12 cA	5.53 ± 0.12 cA
	24	5.66 ± 0.18 bcA	5.76 ± 0.23 bcA	5.93 ± 0.24 cA
	36	6 ± 0.42 bB	6.11 ± 0.67 bAB	6.47 ± 0.42 bA
	48	6.49 ± 0.49 aB	6.69 ± 0.32 aAB	6.97 ± 0.78 aA
Weight Loss Rate (%)	12	7.43 ± 1.21 dA	4.42 ± 0.55 cB	6.47 ± 0.56 cA
	24	12.52 ± 2.26 cA	5.81 ± 0.2 bB	10.95 ± 0.72 bA
	36	17.74 ± 2.12 bA	7.1 ± 0.41 bB	14.05 ± 2.54 bA
	48	23.39 ± 2.12 aA	10.91 ± 0.56 aB	19.69 ± 0.9 aA
TVC (log CFU/g))	0	3.68 ± 0.18 eA	3.68 ± 0.01 eA	3.68 ± 0.01 dA
	12	4.82 ± 0.16 dA	3.97 ± 0.25 dB	3.8 ± 0.16 dB
	24	5.81 ± 0.51 cA	4.52 ± 0.15 cB	4.36 ± 0.36 cC
	36	7.18 ± 0.34 bA	6.38 ± 0.34 bB	6.1 ± 0.34 bC
	48	8.64 ± 0.09 aA	7.51 ± 0.13 aB	7.36 ± 0.67 aC
TVB-N (mg/100 g)	0	6.11 ± 0.31 eA	6.11 ± 0.02 eA	6.11 ± 0.01 eA
	12	9.5 ± 0.24 dA	7.47 ± 0.15 dB	6.5 ± 0.94 dC
	24	14.23 ± 0.36 cA	9.8 ± 0.32 cB	8.51 ± 0.18 cC
	36	21.7 ± 0.05 bA	17.48 ± 0.15 bB	16.95 ± 1.36 bC
	48	26.95 ± 0.36 aA	21.54 ± 1.02 aB	21.06 ± 0.98 aC

Note: Lowercase letters indicate significant differences over time within the same group; uppercase letters denote significant differences among groups at the same time point (*p* < 0.05).

## Data Availability

The original contributions presented in this study are included in the article/Supplementary Materials. Further inquiries can be directed to the corresponding authors.

## Supplementary Materials

The following supporting information can be downloaded at https://www.mdpi.com/article/10.3390/foods14223944/s1, Table S1. Transmittance at 200–800 nm, thickness and opacity of CNF, ChNCs, CNF-ChNCs, and CCBA-x films. Figure S1. Schematic Representation of Hydrogen Bonding Interactions and Final Structure of the Nano-Enhanced Composite Film. Figure S2. The flexibility of the CCBA-9.
