# Supplementary material for: Anthocyanin-Functionalized Hydrophobic Cellulose Composite Films as Sensitive Colorimetric Indicators to Monitor Beef Freshness
Source: Foods. 2025 Nov 18;14(22):3944. doi: 10.3390/foods14223944 (PMC12652354; doi:10.3390/foods14223944)
Supplement: Supplementary file 1 [file foods-14-03944-s001.zip › foods-3960228-SI.pdf]

Supplementary Information

Anthocyanin-Functionalized Hydrophobic Cellulose Composite Films as Sensitive Colorimetric Indicators to Monitor Beef Freshness

Xuemei Cai [1], Changqiu Li [1], Yujie Mo [1], Mingfeng Qiao [1], Jun Xiang [2], Shuang Wang [2,*] and Meifeng Li [3]

1 Cuisine Science Key Laboratory of Sichuan Province, Sichuan Tourism University, Chengdu 610100, China; cxm121517@163.com (X.C.); lcq66988@163.com (C.L.); 18481233522@163.com (Y.M.); mfqiao@163.com (M.Q.)
2 College of Biomass Science and Engineering, Sichuan University, Chengdu 610065, China;
85401296@163.com
3 State Key Laboratory of New Textile Materials and Advanced Processing, Wuhan Textile University,
Wuhan 430200, China; limeifeng2707@163.com
* Correspondence: shuangshine7@scu.edu.cn

Supporting Information

Table S1. Transmittance at 200–800 nm, thickness and opacity of CNF, ChNCs, CNF-ChNCs, and CCBA-x films

Films	Transmittance (%)							Thickness (mm)	Opacity
	200 nm	300 nm	400 nm	500 nm	600 nm	700 nm	800 nm		
CNF	0.11	62.21	76.32	80.44	82.61	83.98	85.08	0.026 ± 0.003	3.19 ± 0.03 g
ChNCs	0.01	3.96	8.13	10.40	11.97	13.06	14.25	0.020 ± 0.001	46.05 ± 0.07 e
CNF-ChNCs	0.04	6.54	12.04	14.98	16.72	18.30	19.65	0.023 ± 0.003	33.78 ± 0.05 f
CCBA-1	0.03	1.25	6.20	7.38	8.32	14.82	17.36	0.021 ± 0.115	51.43 ± 0.08 d
CCBA-3	0.03	0.38	3.77	4.39	4.70	10.70	12.36	0.022 ± 0.002	60.36 ± 0.06 c
CCBA-6	0.03	0.00	1.45	1.00	1.15	9.50	11.86	0.023 ± 0.001	84.34 ± 0.03 b
CCBA-9	0.03	0.02	0.66	0.19	0.30	8.05	10.87	0.023 ± 0.001	109.87 ± 0.09 a

Note: Lowercase letters indicating inter-sample variation, as determined by Tukey's HSD test ($p < 0.05$).

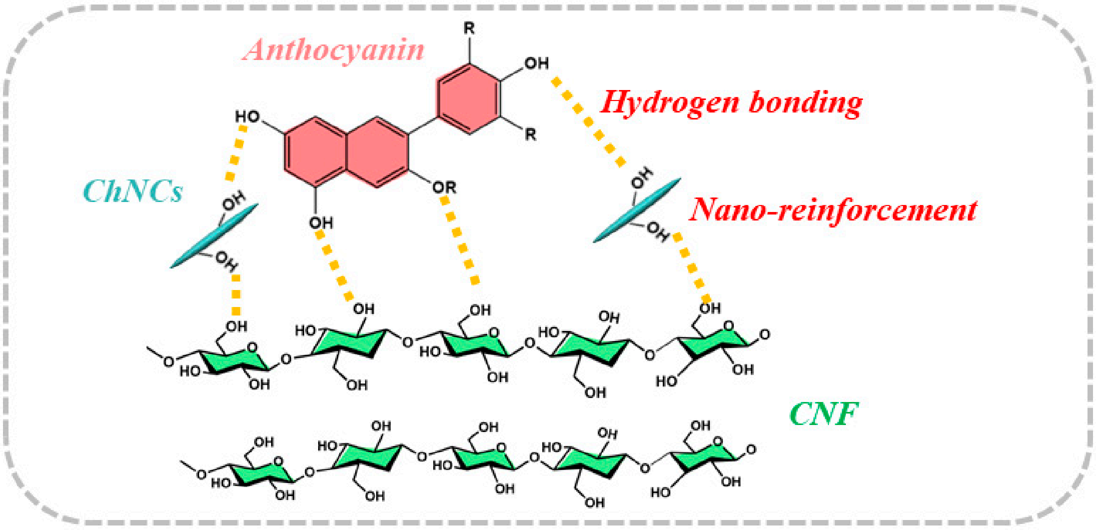

Figure S1. Schematic Representation of Hydrogen Bonding Interactions and Final Structure of the Nano-Enhanced Composite Film.

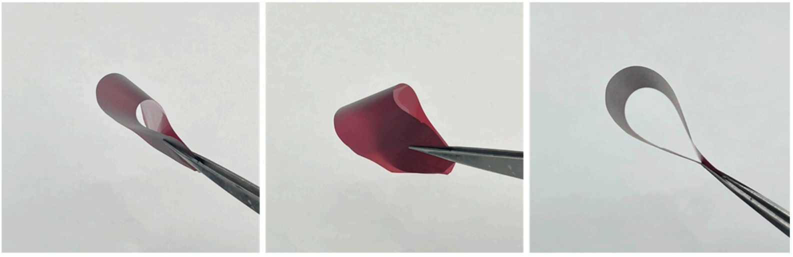

Figure S2. The flexibility of the CCBA-9.
